# Nanopriming of Barley Seeds—A Shotgun Approach to Improve Germination under Salt Stress Conditions by Regulating of Reactive Oxygen Species

**DOI:** 10.3390/plants12020405

**Published:** 2023-01-15

**Authors:** Danuta Cembrowska-Lech, Kinga Rybak

**Affiliations:** 1 Institute of Biology, University of Szczecin, Wąska 13, 71-415 Szczecin, Poland; 2 Molecular Biology and Biotechnology Center, University of Szczecin, Wąska 13, 71-415 Szczecin, Poland

**Keywords:** antioxidants, germination, *Hordeum vulgare*, nanopriming, salt stress

## Abstract

Abiotic stresses are the most important environmental factors affecting seed germination, and negatively affect crop production worldwide. Water availability is essential for proper seed imbibition and germination. The mechanism by which seeds can germinate in areas with high soil salinity is, however, still unclear. The present study aims to investigate the protective roles of AgNPs in alleviating stress symptoms caused by salinity exposure in barley seeds. For this purpose, different treatment combinations of seed priming with PVP-AgNPs in salinity stress conditions were used. Salt stress (150 and 200 mM) was found to reduce seed germination by 100% when compared to the control. Under NaCl concentrations, seed priming with PVP-AgNPs (40 mg L^−1^) only for 2 h, reduced salinity effects. Salinity resulted in increased reactive oxygen species (ROS) generation compared to the control. However, increased antioxidants in the NPs treatments, such as SOD, CAT, GR, GPX (expression at both genes, such as *HvSOD*, *HvCAT*, *HvGR* or *HvGPX*, and protein levels) and glutathione content, scavenged these ROS. Considering all of the parameters under study, priming alleviated salt stress. To summarize, seed priming with AgNPs has the potential to alleviate salinity stress via reduced ROS generation and activation of the antioxidant enzymatic system during germination.

## 1. Introduction

Seed germination is one of the most important stages of the life cycle of higher plants, related to plant survival, reproduction and crop yield. The germination process is divided into three phases of water uptake, and consists of a rapid initial uptake (phase I), followed by a plateau phase (phase II) and a further increase (phase III) [1]. The second phase (II) of water uptake is the most important stage for seed germination, because it determines whether seeds successfully germinate or not, and is accurately regulated by multiple external and internal stimuli. The timing of seed germination is regulated by environmental factors and seeds completely germinate when they are exposed to the appropriate temperature, humidity, light and oxygen. Water plays a key role in seed germination and all necessary metabolic pathways and physiological processes are reactivated during imbibition [1,2]. Salinity affects the germination of seeds and the metabolic processes associated with the first stage of germination by a reduction in seed water absorption, because it induces a variation in the osmotic potential.

Soil salinity, referring to a high concentration of salts in the soil solution, is a wide-ranging issue owing to its adverse impact on agricultural sustainability and environmental health. Salinity problems occur under all climatic conditions and can result from both direct and indirect effects of climate change, as well as human-made interventions, such as unsustainable agricultural practices, improper irrigation management, use of salt rich irrigation water, poor drainage and industrial activities. Saline soils cover an increasing area of the world. Land in the European Union contributes about 3.3% of the global saline soils [3], which is a small region compared to soil salinization hotspots in Central Asia, India, Pakistan, China, Syria, Iraq, Australia and the United States [4].

Increased soil salinization negatively affects most plant growth, due to the high osmotic pressure and cationic/anionic imbalance of the soil solution that makes water uptake by plants difficult. Plant salt tolerance is controlled by different mechanisms, such as the limitation of salt entrance into the plant and avoidance of toxic ion concentrations in the cytosol through the activation of the plasma membrane Na^+^/H^+^ antiporter (SOS1) and the vacuolar Na^+^/H^+^ exchanger (NHX) [5,6]. Salinity also negatively affects seed germination through osmotic effects and ion toxicity [7,8,9]. The inhibition of salinity stress on seed germination is shown by a decreasing germination rate, extended germination time [10], and increasing reactive oxygen species (ROS), resulting in oxidative stress [11]. One of the adaptive mechanisms to contribute to stress tolerance is the activation of ROS scavengers, which modulate redox homeostasis.

Several works in the last decade strongly impart the role of ROS during seed dormancy and germination, not only in metabolically active states but also in quiescent dry states [12,13]. During seed imbibition, their metabolic activity dramatically varies in most, resulting in ROS accumulation [14,15]. Bailly et al. [12] proposed that germination is only completed when the level of ROS is within the ‘oxidative window for germination’, which restricts the start of germination to a critical range of the ROS level, enclosed by lower and higher thresholds. Tight regulation is therefore required to balance ROS production and scavenging for successful seed germination. ROS scavenging systems control the deleterious effects of oxidative stress. During stress, ROS generation can be higher than ROS consumption. ROS signaling promotes transcriptional changes and the reprogramming of cells, which either undergo cell death or cell protection. Therefore, ROS regulation at non-toxic levels could be an important point in the acclimation process of plants to abiotic stress conditions.

Most studies are focused on the possible toxicity of nanoparticles (NPs) on seed germination and plant growth. In the plant life cycle, seed germination is the first step, which determines plant growth in soils polluted with NPs. Different NPs, such as metal or metal oxide, increase or decrease the seed germination of many plant species [16,17]. Therefore, the response of plants to NPs varies depending on the plant species, as well as the type of NPs, molecular size and concentration [18]. Thus, extensive research has sought to precisely define the parameters that affect the final result of NPs on seed germination and plant growth. More recently, nanopriming has emerged as an innovative seed priming procedure with nanoparticles for increasing crop productivity and promoting sustainable agriculture. Important and unique attributes of NPs, such as their surface-area-to-mass ratio, allow them to be efficiently absorbed and delivered substances of interest. For all of the numerous reports on the effect of metal NPs on plant growth parameters, including several physiological and biochemical processes, there is still no clear data on the effect of NPs on seed germination. Because the mechanism of the interaction between NPs and seeds or plants is still not well understood, the final effect of NPs might be both positive or negative. Therefore, intense research on the role of all crucial elements influencing the metal NPs/plant interaction is very important, as it will provide a better understanding of the final outcomes.

Barley (*Hordeum vulgare*) is the world’s fourth most important cereal crop, behind maize, rice and wheat [19]. Moreover, it has several attributes that contribute to its value as a model crop for studies on the mechanisms of stress tolerance, due to its excellent acclimatization to various soil and climatic conditions, including salinization [20]. Investigating the physiological, biochemical and molecular mechanisms could provide global insight into the characteristics of salinity responses in plants, and help to identify key genes involved in plant salinity tolerance. The inhibitory effects of salinity on barley seed germination have been reported, but there is no report regarding the extensive response mechanism to salt stress tolerance induced in the presence of AgNPs during nanopriming. Based on this data, we further explored how AgNPs might alter antioxidant defense systems during barley seed germination under salt stress. It is hypothesized that AgNPs may have potential in promoting seed germination and alleviating salt phytotoxicity. Non-dormant seeds were analyzed during four imbibition time points (before starting the imbibition, one in phase I and two in phase II), comparing the action of AgNPs, which can promote germination in the presence of NaCl. The objectives of this study were to determine: (1) the optimum application concentration of AgNPs; (2); the effects on seed germination indexes; (3) the ROS level; (4) the antioxidative enzymatic activity gene expression and protein level; and (5) the non-enzymatic antioxidants level for half-cell redox potential.

These data provide fundamental insights for future studies regarding the functions of NPs in seed germination under stress conditions. A good understanding of the response mechanisms to salt stress induced in the presence of AgNPs is critical for crop improvement in terms of salt tolerance.

## 2. Results

### 2.1. Effects of AgNPs on Seed Germination under Salinity Stress Conditions

**Figure 1:**
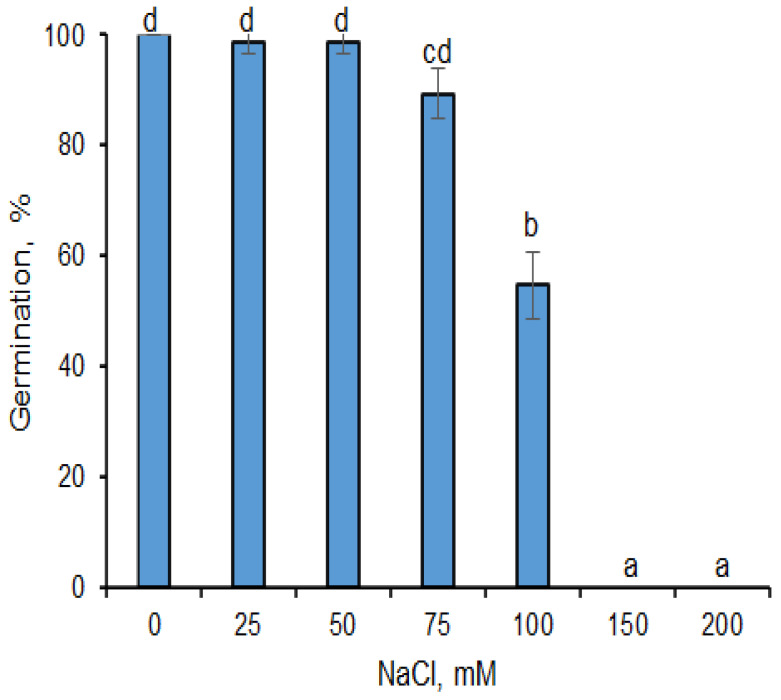
Effect of NaCl on the germination of barley seeds at 25 °C. Results are presented as means ± SD from three replicates of twenty-five seeds each. One-way ANOVA with Duncan’s post hoc test was used to determine the significance of differences. Mean values with different letters (a–d) are significantly different (*p* < 0.05, *n* = 3).

A series of NaCl concentrations (0, 25, 50, 75, 100, 150 and 200 mM) were investigated for their effects on barley seed germination. Results showed that NaCl with a concentration equal to or lower than 75 mM did not affect germination; 90−100% of seeds were germinated (Figure 1). NaCl at a concentration of 100 mM markedly inhibited germination and 45% of seeds were non-germinated. It was also shown that NaCl used at a concentration of 150 or 200 mM totally inhibited the germination of barley seeds and reduced their germination by 100%.

**Figure 2:**
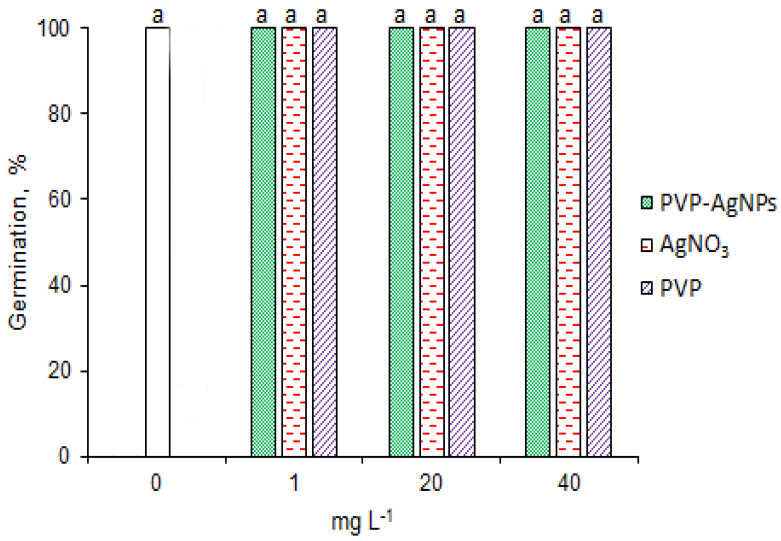
Effect of PVP-AgNPs, AgNO_3_ or PVP on the germination of barley seeds at 25 °C. Results are presented as means ± SD from three replicates of twenty-five seeds each. One-way ANOVA with Duncan’s post hoc test was used to determine the significance of differences. Mean values with different letters are significantly different (*p* < 0.05, *n* = 3).

In order to study the effect of AgNPs on the germination of barley seeds under salinity stress conditions, we employed polyvinylpyrrolidone−coated Ag nanoparticles (PVP−AgNPs) for our analysis. The silver nanoparticles in suspension used in this study were spherical with an average particle size of 30–50 nm and a cubic crystallographic structure. AgNPs at a series of concentrations (0, 1, 20 and 40 mg L^−1^) were investigated for their effects on barley seed germination. The results showed that PVP−Ag−NPs at all concentrations had no effect on germination (Figure 2). In order to investigate the possible effects of Ag^+^ or PVP released from PVP−Ag−NPs, we used 1, 20 and 40 mg L^−1^ AgNO_3_ and 0.02, 40 and 80 µg L^−1^ PVP as the controls, which was equal to the quantity of Ag^+^ or PVP released from PVP−Ag−NPs. No significant effects on seed germination were observed at either AgNO_3_ or PVP concentrations.

**Figure 3:**
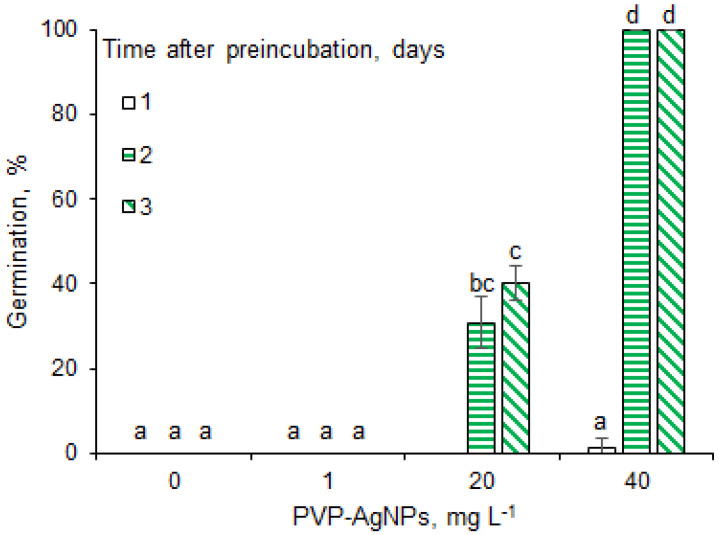
Effect of PVP-AgNPs on the germination of barley seeds in the presence of NaCl at 25 °C. Results are presented as means ± SD from three replicates of twenty-five seeds each. One-way ANOVA with Duncan’s post hoc test was used to determine the significance of differences. Mean values with different letters (a–d) are significantly different (*p* < 0.05, *n* = 3).

To find out if AgNPs overcome the inhibitory effect of NaCl on barley seed germination, PVP−Ag−NPs were used for seed priming before their incubation in the presence of NaCl (150 mM). The results showed that the inhibitory effect of NaCl was completely overcome when seeds were pretreated with AgNPs (Figure 3). Interestingly, the preincubation of barley seeds for only 2 h in the presence of AgNPs was sufficient to completely overcome the inhibitory effect of NaCl. Seeds primed in the presence of AgNPs at a concentration 1 mg L^−1^, did not germinate in the presence of NaCl, but AgNPs at a concentration of 20 mg L^−1^ had a positive effect on germination under salinity stress, with a maximum percentage of germination observed at 40 mg L^−1^.

### 2.2. Effects of AgNPs on ROS Level

Multiple physiological and biochemical mechanisms are expected to be involved in NPs-promoted germination under salt stress conditions. We attempted to determine the adaptive mitigation strategies used by PVP-AgNPs-treated barley seeds under salt stress conditions, particularly the response of ROS and antioxidants (SOD, CAT, GR and GPX) during germination.

**Figure 4:**
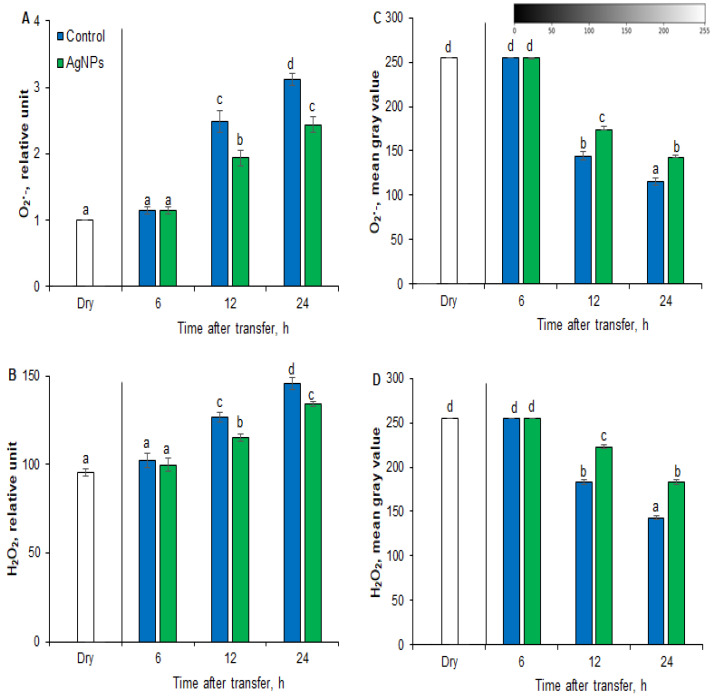
Quantification of O_2_^•−^ (**A**,**C**) and H_2_O_2_ (**B**,**D**) in barley embryos of seeds pre-incubated in water (control) or AgNPs (PVP-AgNPs) for 2 h and transferred to NaCl for different times at 25 °C by flow cytometry (FCM) (**A**,**B**) and image analysis (**C**,**D**). FCM: Fluorescence data are expressed as mean fluorescence intensity (percentage of control). No autofluorescence was present when samples were incubated without dye. Image analysis: Mean gray value of grayscale-converted NBT-stained images, representing differences in staining intensity. Grayscale intensities vary from 0 to 255 (0 = black, 255 = white) with values in between representing shades of gray. Results are presented as means ± SD from five replicates of twenty-five embryos each. One-way ANOVA with Duncan’s post hoc test was used to determine the significance of differences. Mean values with different letters (a–d) are significantly different (*p* < 0.05, *n* = 5).

The aim of the next experiment was to check whether the stimulation of seed germination by AgNPs under salt stress conditions was related to the regulation of ROS content. The superoxide anion (O_2_^•−^) content in the embryos did not change after 6 h of seed imbibition in the presence of NaCl (Figure 4A,C). As the time of imbibition elapsed, the embryos were characterized by 2.5- and 3.3-fold higher content of O_2_^•−^ after 12 and 24 h, respectively, than the embryos from dry seeds. At the same time, embryos from AgNPs-pretreated seeds were characterized by 1.3 times lower content of O_2_^•−^, respectively, than embryos from water-pretreated seeds.

Additionally, the content of hydrogen peroxide (H_2_O_2_) in the embryos did not change after 6 h of imbibition of seeds in the presence of NaCl (Figure 4B,D). After 12 and 24 h from the start of incubation, the embryos were characterized by 1.4- and 1.6-fold higher content of H_2_O_2_, respectively, than the dry seed embryos. At the same time, embryos from AgNPs-pretreated seeds were characterized by 1.3 times lower H_2_O_2_ content than embryos from water-pretreated seeds.

### 2.3. Effects of AgNPs on SOD, CAT, GR and GPX Activity and Gene Expression

The toxic or regulatory role of ROS in cells depends on their concentration. With the increase in ROS levels, antioxidant systems are activated that regulate their concentration in the cell. The enzymatic antioxidant system includes superoxide dismutase (SOD), catalase (CAT), glutathione reductase (GR) or glutathione peroxidase (GPX).

In the previous experiment, it was shown that the preincubation of seeds in the presence of AgNPs reduces the inhibitory effect of NaCl by reducing the ROS level in the embryo. Therefore, it was of interest to check whether AgNPs regulate ROS content by activating the enzymes, such as SOD, CAT, GR and GPX.

**Figure 5:**
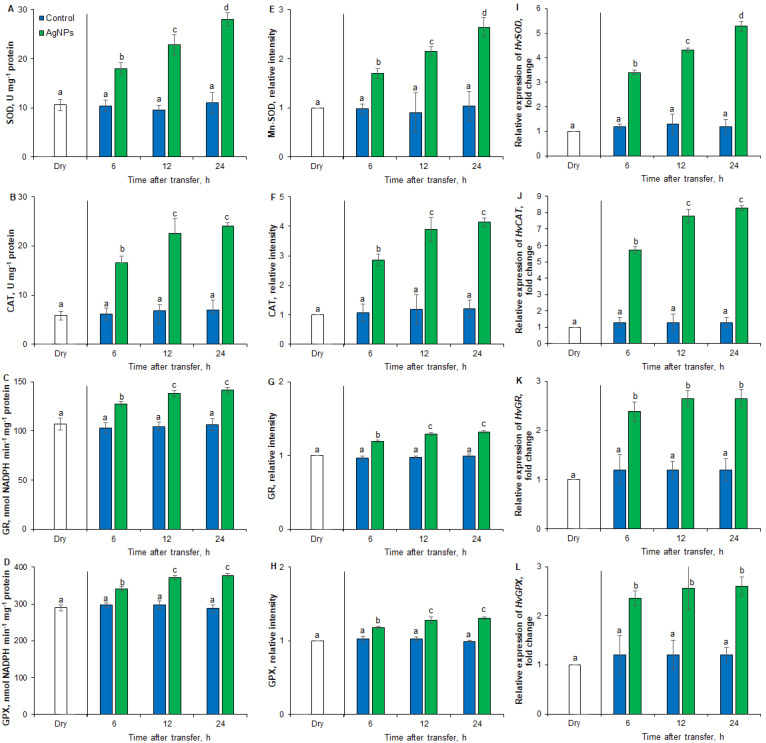
Effect of AgNPs (PVP-AgNPs) on enzyme activity, protein level and gene expression of SOD (**A**,**E**,**I**), CAT (**B**,**F**,**J**), GR (**C**,**G**,**K**) and GPX (**D**,**H**,**L**) in embryo of seeds incubated in the presence of NaCl for different times at 25 °C. Results are presented as means ± SD from five replicates of twenty-five embryos each. For analysis of enzymes activities and protein levels, one-way ANOVA with Duncan’s post hoc test was used to determine the significance of differences. For gene expression analysis, the fold changes indicate the expression patterns of analyzed genes relative to their transcript levels in the embryo from dry seeds with an assumed value of 1; one-way ANOVA with Tukey’s (HSD) post hoc test was used to determine the significance of differences. Mean values with different letters (a–d) are significantly different (*p* < 0.05, *n* = 5).

The activity of SOD, a metalloenzyme catalyzing the superoxide anion dismutation, in embryos did not change during 24 h of seed incubation in the presence of NaCl (Figure 5A). AgNPs applied for 2 h resulted in a 1.7-, 2.4- and 2.6-fold increase in SOD activity after 6, 12 and 24 h of imbibition in the presence of NaCl, respectively. It was also shown that both embryos from dry seeds and those incubated in the presence of NaCl were characterized by the presence of the Mn-SOD protein (Figure 5E). AgNPs applied for 2 h increased the level of Mn-SOD in embryos from seeds incubated in salt stress conditions for 6, 12 and 24 h by 1.7, 2.4 and 2.6 times, respectively, compared to embryos from water-pretreated seeds.

Since SOD activity is one of the sources of hydrogen peroxide formation, in the next experiment, it was decided to check whether the stimulating effect of AgNPs on seed germination under salt stress conditions was related to the activity of catalase (CAT), an enzyme catalyzing the two-stage reaction of hydrogen peroxide dismutation to H_2_O and O_2_. CAT activity in the embryos either did not change or only slightly changed during the 24-h imbibition of seeds in the presence of NaCl. (Figure 5B). A 2.7–3.5-fold increase in CAT activity was found in embryos from AgNPs-pretreated seeds, compared to embryos from water-pretreated seeds. It was also shown that both embryos from dry seeds and those incubated in the presence of NaCl were characterized by the presence of the CAT protein (Figure 5F). AgNPs applied for 2 h increased CAT levels in embryos from seeds incubated in salt stress conditions for 6, 12 and 24 h by 3, 3.3 and 3.5 times, respectively, compared to embryos from seeds preincubated in the presence of water.

The aim of the next experiment was to assess the effect of AgNPs on the activity of the glutathione reductase (GR) and glutathione peroxidase (GPX), which regulate the content of glutathione in the oxidized (GSSG) and reduced (GSH) forms. GR and GPX activity did not change during the 24-h seed imbibition in the presence of NaCl (Figure 5C,D). The embryos from seeds preincubated in the presence of AgNPs were characterized by a 1.4-fold higher activity of both enzymes during incubation in the presence of NaCl for 6, 12 and 24 h, respectively, compared to embryos from water-pretreated seeds. It was also shown that both dry seeds and seeds incubated in the presence of NaCl were characterized by the presence of the GR (Figure 5G) and GPX (Figure 5H) proteins. AgNPs applied for 2 h increased 1.3 times the GR and GPX levels in embryos during 24 h of salt stress conditions, compared to embryos from water-pretreated seeds.

To find out the molecular basis of antioxidant defense system involvement in the induction of germination in barley seeds under salinity stress conditions due to the application of AgNPs, the expression levels of *HvSOD*, *HvCAT*, *HvGR* and *HvGPX* genes were assessed (Figure 5I–L). When compared to embryos from dry seeds, the level of *HvSOD*, *HvCAT*, *HvGR* and *HvGPX* transcripts in embryos from water-pretreated seeds remained unchanged during the whole period of incubation. In comparison, present genes were upregulated when seeds were pretreated with AgNPs. The expression of the *HvSOD* gene showed a nearly 4.5-fold induction of its transcriptional activity when seeds were incubated for up to 24 h in the presence of NaCl (Figure 5I). About a 4.5-fold change in the transcript level was observed for *HvCAT* at 6h of incubation of NPs-pretreated seeds, with a peak in the transcript level at 24 h of incubation; a 6.5-fold change in the transcript level was observed (Figure 5J). In comparison, the expression of both *HvGR* and *HvGPX* genes showed nearly a two-fold induction up to 24 h of incubation (Figure 5K,L).

### 2.4. Effets of AgNPs on Glutathione Level and Half-Cell Redox Potential

It has been shown that the induction of barley seed germination by AgNPs under salt stress conditions is associated with an increase in GR and GPX activity in embryos (Figure 5C,D,G,H). Literature data indicated that ROS affects the redox potential in cells; e.g., by affecting the change in the content of GSH and GSSG [12].

**Figure 6:**
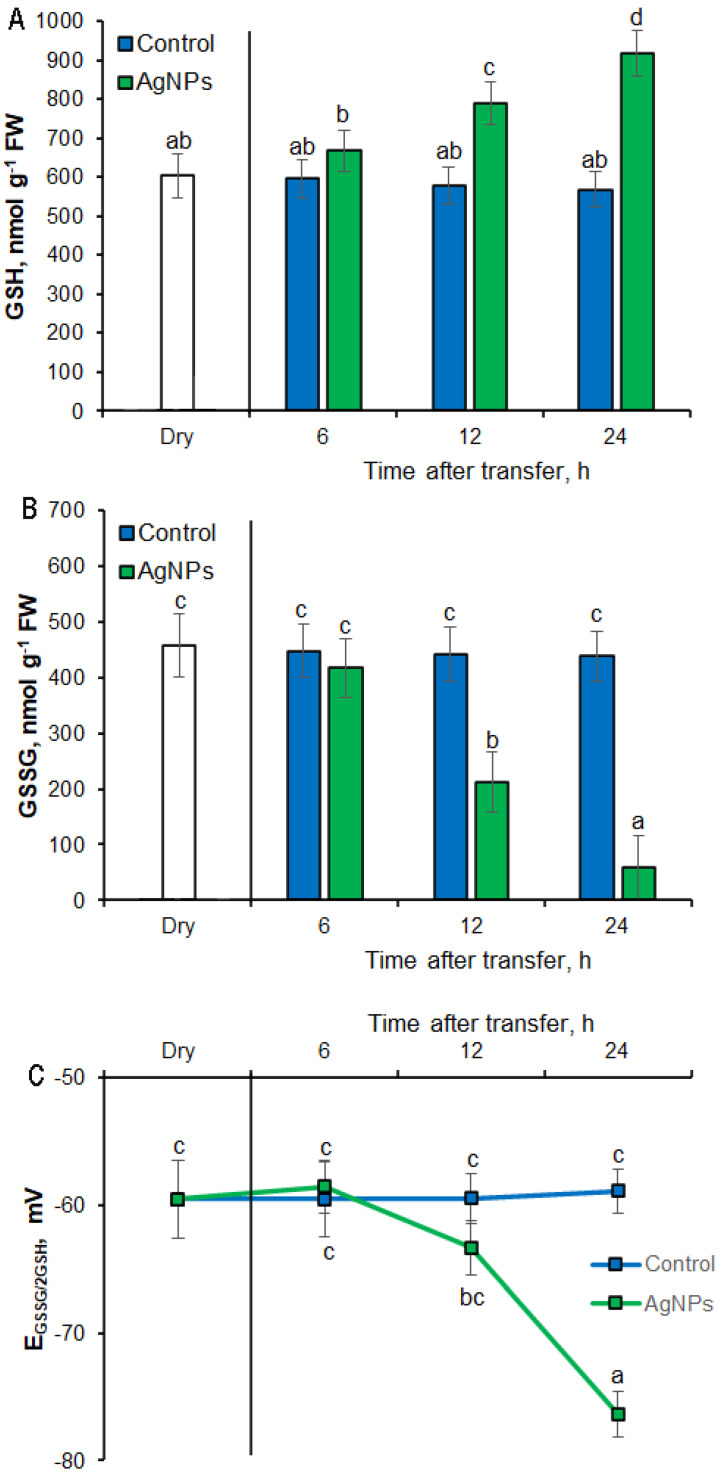
Quantification of GSH (**A**), GSSG (**B**) and reduction potential (**C**) in the barley embryo of seeds pre-incubated in water or AgNPs (PVP-AgNPs) for 2 h and transferred to NaCl for different times at 25 °C. Results are presented as means ± SD from five replicates of twenty-five embryos each. One-way ANOVA with Duncan’s post hoc test was used to determine the significance of differences. Mean values with different letters (a–d) are significantly different (*p* < 0.05, *n* = 5).

The levels of GSH and GSSG in the embryos did not change during 24-h incubation of the seed in the presence of NaCl (Figure 6A,B). AgNPs significantly increased (1.4-fold) GSH content in the embryos and decreased GSSG content 2-fold, compared to embryos from water-pretreated seeds, during 12 h of imbibition in salt stress conditions. After 24 h of incubation, embryos from AgNPs-pretreated seeds were characterized by the highest GSH content and the lowest GSSG content, compared to embryos from water-pretreated seeds; the embryos were then characterized by 1.7 times higher content of GSH and 8 times lower content of GSSG. It has also been shown that the presence of the glutathione pool in embryos is essential to maintain redox balance. The reduction potential (EGSSG/2GSH) in the embryo cells did not change during the 24-h incubation in the presence of NaCl and was −0 mV (Figure 6C). AgNPs caused a significant decrease in the redox potential after 24 h of imbibition in salt stress conditions; the redox potential was then −80 mV.

## 3. Discussion

One of the most serious environmental problems confronting agricultural production today is abiotic stress, which drastically reduces the amount of land planted and results in significant crop losses worldwide. Salinity is one of the most pressing abiotic stressors. Saline conditions are rapidly increasing, along with the alarming rise of global warming [21]. Seed germination and early seedling growth are the most sensitive stages to water limitation in many crops, resulting in delayed germination and lowering its rate and uniformity, thus resulting in poor crop performance and yield [22,23].

Soil salinity inhibits seed germination due to the low osmotic potential of soil solution generated around the seeds, which obviates water imbibition [24,25,26,27]. In this study, salt stress conditions (150 mM NaCl) completely decreased the germination of barley seeds (Figure 1). Reducing germination ability by increasing salinity has been described by many researchers [28,29] and is probably the effect of disruptions by various biochemical reactions in the cells [30]. In addition to this, some authors have described that decreased germination may also be due to a reduced water potential in seed embryos and a delay in the breakdown of biomolecules in the storage tissue, such as endosperm [31].

Seed vigor is critical for seedling establishment and tolerance to hostile environments. Tolerating abiotic stress by seedlings more uniformly requires a robust seed with enhanced seed vigor, which includes rapid germination. Seed priming induces a specific physiological state in plants by applying natural and synthetic chemicals to the seed prior to germination. Thus, seed priming may be an effective approach to develop stress tolerance in plants [32]. The nanopriming technique is an effective strategy that uses less nanoparticles (NPs), has lower costs and reduces the risk of nanomaterials harming the environment, and thus may be a good candidate for improving seed tolerance to salinity stress [33]. In comparison to classic seed priming, in nanopriming, seeds are imbibed in nanosuspensions, and the NPs may or may not be taken up by the seeds [34] and the majority remain on the seed surface as a coating [35,36].

Previous studies have shown that the seed germination of many species, such as *Zea mays*, *Triticum aestivum*, *Oryza sativa* and *Lactuca sativa*, depends on the dose of NPs, and seedling growth is the result of physical and biochemical responses to NPs, such as water absorption activity, enzymatic activity, expression of specific genes, antioxidants activity and metabolism of storage substances [37,38,39,40,41]. This study shows that priming barley seeds with AgNPs at a 40 mg L^−1^ concentration for 2 h at 25 °C completely reversed the negative effects of salt stress and ameliorated germination parameters to an appreciable amount during salinity stress. This effect could be obtained due to the better penetration of AgNPs into seed pores [30], which increased the efficiency of water uptake, resulting in a notable acceleration in the germination of barley seeds. This is confirmed by literature data, indicating that nanopriming accelerates water uptake by seeds and enables earlier radicle rupture through the covered tissue, compared to conventional methods of seed priming. For example, AgNPs (10 ppm) improved the germination rate of *Oryza sativa* seeds, and seeds treated with AgNPs absorbed more water during imbibition [42]. Similarly, nanopriming with cerium oxide nanoparticles coated with poly-(acrylic) acid (PNC, at a concentration 0.1 mM), improved water imbibition and germination of *Brassica napus* in salt stress conditions [33]. Another study reported that *Triticum aestivum* nanopriming with ZnO-NPs increased germination and shoot height, and improved overall plant physiology, including growth under salt stress conditions [43].

High salinity leads to excessive production and accumulation of ROS, such as O_2_^•−^ and H_2_O_2_, affecting seed germination and subsequent seedling growth. Multiple biochemical mechanisms are expected to be involved in AgNPs-promoted germination under salt stress conditions. We attempted to determine the biochemical mechanisms used by AgNPs-treated seeds under salinity stress, particularly the response of ROS and antioxidants (SOD, CAT, GR, GPX, glutathione) during germination. Usually, a balance between ROS and antioxidants is required for seed germination. In the present study, a 150 mM NaCl concentration causes a significant increase in ROS generation due to oxidative stress (Figure 4). AgNPs-primed seeds, on the other hand, showed reduced oxidative stress to decrease ROS content in barley seeds under salt stress conditions. Under salinity stress, similar results were observed in PNC nanopriming seeds of *Gossypium hirsutum* and *Brassica napus* [33,44].

Nanoparticles have been shown to effect antioxidant responses during seed germination and plant growth under salt stress conditions. In our experiment, biochemical stress markers, such as enzymatic and non-enzymatic antioxidants, were enhanced with high intensity during salt stress conditions. Enzymatic antioxidants, such as SOD, CAT, GR and GPX, act as the first line of defense in stress-induced responses, while non-enzymatic antioxidants, such as glutathione (GSH, GSSG), are mainly regarded as components of the redox homeostasis system. Since these parameters are only acquired during stressful cues, an increase in antioxidant activities under NaCl and a significant reduction in response of AgNPs satisfy the considerate physiological responses in accordance with the given treatment by maintaining the homeostatic equilibrium. In the present study, higher antioxidant enzyme activity (SOD, CAT, GR and GPX) and non-enzymatic antioxidant (glutathione) levels were observed when barley seeds were primed with AgNPs (Figure 5 and Figure 6), which was further confirmed by the upregulation of the relative gene expression of *HvSOD*, *HvCAT*, *HvGR* and *HvGPX* (Figure 5). SOD is the primary enzymatic antioxidant that catalyzes the detoxification of O_2_^•−^, into H_2_O_2_ and molecular oxygen. This results in an increase in the H_2_O_2_ concentration inside the cell, but CAT converts it into H_2_O and O_2_. Recent studies have shown that nanopriming helps plants to maintain ROS homeostasis by increasing antioxidant activity in salt stress conditions. Ye et al. [45] observed that MnSOD was upregulated in MnNPs-primed seeds of *Capsicum annuum* L., which enhanced the SOD enzyme levels by upregulating the relative expression level of *Mn-SOD* genes. Seed priming with AgNPs and TiO_2_NPs improved SOD, POD and CAT in *Solanum Lycopersicum* and *Zea mays*, respectively [46,47]. Kumar et al. [48] reported that AgNP priming enhanced antioxidant enzymes, such as SOD, POD, CAT and APX, in *Psophocarpus tetragonolobus* L. Furthermore, nanopriming with cerium oxide nanoparticles coated with poly (acrylic) acid (PNC) improved cotton salt tolerance by maintaining ROS homeostasis; PNC nanopriming increased the activities of SOD and POD to scavenge ROS during seed imbibition. Similarly, PNC nanopriming improved cotton salt tolerance through the effective regulation of the gene expression of ROS enzymatic pathways, such as GST, POD and PRX families.

The glutathione pool and the GSH/GSSG ratio play important roles in redox signaling during seed germination. The non-enzymatic antioxidants, GSH and GSSG, together with enzymes of the glutathione cycle, can also help coordinate tolerance to salt stress conditions. However, the relative participation of GSH metabolism in nanoprimed seeds is not fully understood. We studied the glutathione pool, in both reduced and oxidized forms, and the GSH/GSSG ratio in barley nanoprimed seeds, focusing on redox changes in the embryo during germination under salt stress conditions. In the present study, for the first time, we indicated changes in the GSH/GSSG redox that occur in nanoprimed seeds. We suggest that EGSSG/2GSH can be used as markers of the physiological stage of nanoprimed seeds under stress conditions. We also present a model of interaction between GSH and GSSG with ROS in the redox regulation of nanoprimed seeds under salt stress conditions.

## 4. Materials and Methods

### 4.1. Plant Material

Barley (*Hordeum vulgare* L.) seeds were collected in June 2018 in crop fields near Szczecin (Poland). Experiments were carried out with non-dormant grains (grains from the same harvest but stored for 6 months at 23 °C) that had been stored at −20 °C. In the experiments, only dehulled grains were used so both lemma and palea (glumellae) were removed by hand.

### 4.2. Ag Suspensions

PVP−coated Ag nanoparticles (PVP−AgNPs) were purchased as a dry powder (Nanoamorphous Materials, Los Alamos, NM, USA). The characteristics of nanoparticles were: the spherical morphology of particles and a cubic crystallographic structure. Dry silver nano powder (Ag, 99.9%, w/0.2 wt% PVP) was suspended in ultrapure water to make a 250 mg Ag L^−1^ stock suspension by sonicating them for 10 min. AgNO_3_ was prepared in ultrapure water and used to compare the toxicity of AgNPs.

### 4.3. Germination Assay and Treatment Experiments

In all experiments, seeds (25 in each of the 3 biological replicates per treatment) were incubated at 25 °C in darkness, in Petri dishes (ø 6 cm) on one layer of filter paper (Whatman no. 1) moistened with 2.0 mL of deionized water or a solution. Germinated seeds were counted every day up to the fifth day of incubation. Seeds were considered as germinated when the radicle protruding through the coleorhiza was above 1 mm in length [49].

To determine the effect of AgNPs on seed germination under salinity stress, seeds were incubated for 2 h in 2.0 mL of deionized water or 1, 20 or 40 mg L^−1^ of Ag of either PVP−AgNPs or AgNO_3_, and 0.02, 40 or 80 µg L^−1^ of PVP at 25 °C. Subsequently, seeds were rinsed with sterile deionized water and were transferred to Petri dishes (ø 6 cm) on one layer of filter paper (Whatman no. 1) moistened with 2.0 mL of distilled water or NaCl solution and incubated in the same conditions for up to 5 days.

### 4.4. Superoxide Anion and Hydrogen Peroxide Content

ROS content in embryos (25 in each of the 5 biological replicates per treatment) was determined according to the procedure described by Cembrowska-Lech [50] using dihydroethdium (DHE) (ThermoFisher, Waltham, MA, USA) for O_2_^•−^, and CDCDHFDA-AM (6-carboxy-2’,7’-dichlorodihydrofluorescein diacetate) (ThermoFisher, Waltham, MA, USA) for H_2_O_2_ analysis. The labeled cells were analyzed using a flow cytometer (Partec) with an air-cooled 20 mV argon-ion laser. The final gated cell populations contained 20,000 cells and signals were recorded on a histogram by logarithmic amplifiers. The histograms presented the fluorescence intensity (log; Geo Mean) on the *x*-axis and cell count on the *y*-axis in a gated population of cells. The relative O_2_^•−^ and H_2_O_2_ levels were expressed as the mean fluorescence intensity (percentage of the control).

The generation of ROS in situ was detected by monitoring the reduction of nitro blue tetrazolium (NBT) for O_2_^•−^ as described by Beyer and Fridovich [51] or by polymerization of 3,3’−diaminobenzidine (DAB) for H_2_O_2_ according to Thordal−Christensen et al. [52]. After incubation, embryos (25 in each of the 5 biological replicates per treatment) were dissected and stained for 10 min in 6 mM NBT (in 10 mM Tris−HCl, pH 7.4) or for 90 min in darkness in 1 mg/mL DAB containing 0.05% (*v*/*v*) Tween−20 and 10 mM Na_2_HPO_4_. Dark blue staining in the presence of NBT indicated O_2_^•−^ production or yellow staining in the presence of DAB indicated polymerization of DAB, requiring H_2_O_2_ and peroxidase activity. After removing the staining solution and rinsing the embryo three times in sterile water, the embryo was photographed (Canon EOS 500). All of the images were saved as TIFF files, with 3072 × 2304 pixel resolution and 24-bit RGB color depth (pixel transformation factor = 1, no scaling of result images). Image analysis was conducted using Fiji ImageJ [53]. NBT- or DAB-stained images were converted to grayscale for analysis. Minimum and maximum threshold values were established to remove background staining and a mean gray value for the plaques was then calculated [50]. The data were presented as a mean gray value of the total plaque area to give a relative staining density for each sample.

### 4.5. Enzymes Extraction and Assays

All enzyme assays were performed in the same crude extract. After incubation, the embryos were isolated from the seeds (25 in each of the 5 biological replicates per treatment), immediately frozen in liquid N_2_ and stored at −80 °C prior to enzyme assay. Samples were ground to a fine powder in liquid N_2_ using a Retsch MM200 (Haan, Germany) laboratory mill ball and homogenized for 10 min in 0.1 M potassium phosphate buffer (pH 7.0) containing 10 mM ethylenediaminetetraacetic acid (EDTA) and 1% (*w*/*v*) PVP (fresh weight: buffer, 1:10, *w*/*v*). Homogenates were centrifuged for 20 min at 15,000× *g* at 4 °C.

Superoxide dismutase (EC 1.15.1.1) activity was tested according to Giannopolitis and Ries [54] by the inhibition of NBT chloride photoreduction. The assay was carried out using the following reaction mixture: 0.1 M potassium phosphate buffer (pH 7.8), 1.3 µM riboflavine, 13 mM methionine, 63 µM NBT, 0.1 mM EDTA and 100 µL of the enzymatic extract. The reaction mixture was illuminated (50 µmol m^−2^ s^−1^) at 25 °C for 10 min and the absorbance was measured at 560 nm. One unit of SOD activity was defined as the amount of the enzyme required to inhibit the reduction of NBT by 50% under the specified conditions. SOD activity of the extracts was expressed as U mg^−1^ protein.

Catalase (EC 1.11.1.6) activity was measured according to Rao et al. [55]. Enzyme activity was spectrophotometrically monitored at 240 nm for 60 s using the following mixture: 50 mM potassium phosphate buffer (pH 7.0), 14.3 mM H_2_O_2_ and 100 µL of enzymatic extract. Purified CAT was used as a calibration standard. CAT activity was expressed as U mg^−1^ protein.

Glutathione reductase (EC 1.8.1.7) activity was analyzed as described by Esterbauer and Grill [56] by following the rate of NADPH oxidation at 340 nm for 3 min. The assay mixture contained: 0.1 mM potassium phosphate buffer (pH 7.8), 0.5 mM NADPH, 10 mM oxidized glutathione (GSSG), 10 mM EDTA and 100 µL of enzyme extract. The GR activity was expressed as nmol NADPH min^−1^ mg^−1^ protein.

Glutathione peroxidase (EC 1.11.1.9) activity was conducted as described by Nagalakshmi and Prasad [57] by following the rate of NADPH oxidation at 340 nm for 5 min. The reaction mixture contained: 0.5 M potassium phosphate buffer (pH 8.2), 10 mM EDTA, 1.14 M NaCl, 10 mM GSH, 2 mM NADPH and 2.5 mM H_2_O_2_, and 100 µL of enzyme extract. The reaction was started by adding 2 U of GR. GS activity was expressed as nmol NADPH min^−1^ mg^−1^ protein.

### 4.6. Protein Assay

The protein content in the enzymatic extracts was assayed using Bradford’s method [58], using bovine serum albumin (BSA) as a standard.

### 4.7. Western Blot Analysis

After incubation, the embryos (25 in each of the 5 biological replicates per treatment) were dissected, immediately frozen in liquid N_2_ and stored at −80 °C prior to enzyme assay. All samples were ground to a fine powder in liquid nitrogen using a Retsch MM200 (Haan, Germany) laboratory mill ball and homogenized in the lysis buffer containing 62.5 mM Tris–HCl (pH 6.8), 2% (*w*/*v*) SDS, 15 mg/mL DTT and 7% (*v*/*v*) glycerol. After homogenization, the samples were boiled for 10 min and centrifuged for 20 min at 15,000× *g*. Samples containing 50 µg protein were loaded per line and separated on 12% SDS-PAGE gel following Laemmli [59]. After electrophoresis, the gels were electroblotted onto PVDF membranes (Millipore, Burlington, MA, USA). Following triple washing in TBST, the blotting membranes were incubated in a blocking solution and probed with the polyclonal antibody: CAT (AS09 501, Agrisera, Vännäs, Sweden), MnSOD (AS09 524, Agrisera, Vännäs, Sweden)**,** GR (AS06 181, Agrisera, Vännäs, Sweden) and GPX (AS06 183, Agrisera, Vännäs, Sweden). The membranes were then washed three times in TBST and probed with peroxidase conjugated secondary antibody (AS09 602 or AS09 603, Agrisera, Vännäs, Sweden). The immunoblots were incubated with a detection solution containing acetate buffer, diaminobenzidine and H_2_O_2_. The data were represented as immunoblot band visualization and the band intensities were determined using the Fiji ImageJ software v2.9.0 [53].

### 4.8. Determination of Glutathione Content and Glutathione Half-Cell Reduction Potential

Glutathione in the reduced (GSH) and oxidized (GSSG) form was assayed following Smith [60]. After incubation, the embryos were isolated from seeds (25 in each of the 5 biological replicates per treatment), immediately frozen in liquid N_2_ and stored at −80 °C prior to glutathione assay. Twenty-five embryos were ground to a fine powder in liquid N_2_ using a Retsch MM200 (Haan, Germany) laboratory mill ball. The ground samples were extracted in 1 mL of ice cold 5% (*w*/*v*) sulphosalicylic acid (fresh weight: sulphosalicylic acid, 1:10, *w*/*v*) and centrifuged at 10,000× *g* for 20 min at 4 °C. A 1 mL aliquot of the supernatant was neutralized by adding 1.5 mL of 0.5 M potassium phosphate buffer (pH 7.5) and used to measure total glutathione (GSH + GSSG). Another 1 mL of the neutralized supernatant was pretreated with 0.2 mL of 2-vinylpyridine for 1.5 h at 25 °C to mask GSH and to allow determination of GSSG alone. Both samples were extracted twice with 5 mL of diethylether. The incubation mixture contained: 0.5 mL of 0.1 M sodium phosphate buffer (pH 7.5) with 5 mM EDTA, 0.2 mL of 5 mM 5,5’-dithiobis-(2-nitrobenzoic acid), 0.1 mL of 2 mM NADPH, 0.1 mL of glutathione reductase type III and 0.1 mL of extract. The change in absorbance at 412 nm was followed at 25 °C. A standard curve was prepared using the GSH standard. The amount of reduced glutathione was calculated as the difference between the total and the oxidized glutathione. The results are expressed as nmol GSH g^−1^ FW and as nmol GSSG g^−1^ FW.

Calculation of E_GSSG/2GSH_ following the formulas given in Schafer and Buettner [61] using the Nernst equation:

$$E_{\mathrm{GSSG}/2\mathrm{GSH}}=E^{0^{\prime }}-\frac{\mathrm{RT}}{\mathrm{nF}}\ln\frac{{\left[\mathrm{GSH}\right]}^{2}}{\left[\mathrm{GSSG}\right]}$$

where R is the gas constant (8.314 J K^−1^ mol^−1^); T, the temperature in K; n, the number of transferred electrons; F, the Faraday constant (9.6485 × 10^4^ C mol^−1^); E^0^’, standard half-cell reduction potential at pH 7 [−240 mV]; [GSH] and [GSSG] are molar concentrations of GSH and GSSG. The density of water, approximated as 1 g ml^−1^, and the amount of water per gram of seed were used in the calculations of molar concentrations of GSH and GSSG.

### 4.9. RNA Extraction, cDNA Synthesis and RT-qPCR

To estimate SOD, CAT, GR and GS genes expression levels, total RNA was extracted from *H. vulgare* embryos isolated from seeds (25 in each of the 5 biological replicates per treatment) at different time-points of incubation (0, 6, 12 and 24 h), immediately frozen in liquid N_2_ and stored at −80 °C until RNA extraction.

The RNA was conducted as described by Oñate-Sánchez and Vicente-Carbajosa [62] for tissues with a high content of polysaccharides, with some modifications. Twenty-five embryos were ground to a fine powder in liquid N_2_ using a Retsch MM200 (Haan, Germany) laboratory mill ball and ca. 80 mg of powdered tissue was used for RNA extraction.

DNAse I (Ambion)) was used for the removal of genomic DNA from RNA samples. Reverse transcription of RNA into cDNA was performed using the High-Capacity cDNA Reverse Transcription Kit (ThermoFisher, Waltham, MA, USA) according to the manufacturer’s protocol. The amplification reactions were performed using HOT FIREPol EvaGreen qPCR Mix Plus (ROX) (Solis BioDyne, Tartu, Estonia) on a Real-Time PCR System (BioRad, Hercules, CA, USA). qPCR reactions were performed in triplicate for each of the three biological replicates per treatment. A BLAST search was performed against the *H. vulgare* databases of Plant GDB (Plant Genome Database, http://www.plantgdb.org, 15 December 2011 ) and NCBI (National Center of Biotechnology Information, http://www.ncbi.nlm.nih.gov). The primers used in RT-qPCR analyses are listed in Table S1. Relative values of transcripts were calculated by normalizing against the amount of mRNA for a *HvActin* [63] or *Actin* SGN-U580609, or following the Pfaffl [64] method. The quantitative PCR values were compared with zero time (dry seed) for each time point and expressed as relative levels of expression.

### 4.10. Statistical Analysis

All of the experiments were carried out in three or five biological replicates and the results were expressed as mean ± SD. The means were analyzed for significance using one–way analysis of variance, ANOVA (Statistica for Windows v. 13.0, Stat–Soft Inc., Tulsa, OK, USA). Data were checked for normality and homogeneity of variance, and met these criteria. Duncan’s multiple range test was used to test for significance of differences (*p* ≤ 0.05) in germination experiments, ROS level and enzymatic activity assays. Statistical analyses of gene expression were performed using a post hoc Tukey’s (HSD) test with a confidence interval of 0.05 and a confidence level of 95%. Differences between the mean values were considered to be significant at *p* < 0.01 or *p* < 0.05. Every experiment was repeated three times and the results presented correspond to a representative single experiment.

## 5. Conclusions

Nanopriming with AgNPs of barley seeds is a promising field for exploitation in the agricultural industry, as determined by the results presented in this research paper. Considering food insecurity mediated by climate change, such applications could prove to be beneficial in the future. Seed priming with AgNPs is helpful in salt stress mitigation by modulating anti-oxidant defense systems. In addition to the improvement of the maintenance of ROS homeostasis, it will also be important to understand the effect of AgNPs nanopriming on the ability to maintain the Na+/K+ ratio for understanding the mechanism underlying better salt tolerance in nanoprimed seeds. The current study recommends priming with AgNPs for barley seeds as a pre-sowing treatment. However, further experimentation in the field is necessary and it is recommended to explore the potential of nanoseed priming in other crops as well. However, it remains unclear whether the AgNPs nanopriming improvement in terms of salt tolerance could last until plant harvest or not. Further comprehensive studies are required for field-scale applications of AgNPs in saline soils.

## Data Availability

Not applicable.

## Supplementary Materials

The following supporting information can be downloaded at: https://www.mdpi.com/article/10.3390/plants12020405/s1, Table S1: Primers of genes coding for antioxidant enzymes used in qRT-PCR.
