# Supplementary material for: Nanopriming of Barley Seeds—A Shotgun Approach to Improve Germination under Salt Stress Conditions by Regulating of Reactive Oxygen Species
Source: Plants (Basel). 2023 Jan 15;12(2):405. doi: 10.3390/plants12020405 (PMC9864488; doi:10.3390/plants12020405)
Supplement: Supplementary file 1 [file plants-12-00405-s001.zip › plants-2125476-supplementary.pdf]

Table S1. Primers of genes coding for antioxidant enzymes used in qRT-PCR.

**Target gene (accessions)**	**Forward primer 5′– 3′ sequence**	**Reverse primer 5′– 3′ sequence**
HvSOD (AK252295)	CCGAAGATGAAATCCGCCAT	GCCTCCTGATCCATGGCGCCGAC
HvCAT (U20778)	CCATAAGGGACCATGCATGCCAGCTAC	GCTCGAAGCACCCACTTTAGTTTAAGC
HvGR (AK376974)	TGGAGGCTACTTGCTTTGCT	AGATAGCGGCGGAATACAGA
HvGPX	CTGATCCCGCACTACAGCTT	CCCGTCCTTGAGCATCTTCC
Hvactin (AY145451)	GCCGTGCTTTCCCTCTATG	GCTTCTCCTTGATGTCCCTTA
